# Overcoming disruptions in essential health services during the COVID-19 pandemic in Mexico

**DOI:** 10.1136/bmjgh-2021-008099

**Published:** 2022-03-08

**Authors:** Svetlana V Doubova, Zoé Alejandro Robledo-Aburto, Célida Duque-Molina, Gabriela Borrayo-Sánchez, Margot González-León, Ricardo Avilés-Hernández, Saúl Eduardo Contreras-Sánchez, Hannah H Leslie, Margaret Kruk, Ricardo Pérez-Cuevas, Catherine Arsenault

**Affiliations:** 1 Unidad de Investigación Epidemiológica y Servicios de Salud del CMN Siglo XXI, Instituto Mexicano del Seguro Social, Ciudad de México, México; 2 Dirección General, Instituto Mexicano del Seguro social, Ciudad de México, México; 3 Division of Prevention Science, University of California San Francisco, San Francisco, California, USA; 4 Department of Global Health and Population, Harvard University T H Chan School of Public Health, Boston, Massachusetts, USA; 5 Division of Social Protection and Health, Inter American Development Bank Jamaica Country Office, Kingston, Jamaica

**Keywords:** COVID-19, health systems, health policy

**Summary box**

- Significant disruptions in health services during the first year of the COVID-19 pandemic prompted the Mexican Institute of Social Security (IMSS) to implement the National Strategy for Health Services Recovery (NHSR strategy) to ensure resumption of essential health services provided to almost 68 million IMSS affiliates.
- Starting in April 2021, the NHSR strategy included six major components: (1) reconversion of repurposed COVID-19 hospitals; (2) strengthening COVID-19 preventive measures; (3) adjusting governance to prioritise essential health services and enhance service delivery, including optimisation of family medicine clinics and hospital working shifts and nationwide weekend services delivery and monothematic (focused on one service or disease) healthcare days; (4) implementation of telemedicine services; (5) reinforcement of preventive services and health promotion activities and (6) regular monitoring of essential health services.
- Lessons learnt could be used as an opportunity to strengthen and modernise IMSS healthcare and enable the institution to satisfy the demand and health needs of the population.
- Future research should assess whether the NHSR strategy was successful in bringing service levels back to prepandemic trends and overcoming the backlogs in care to inform continued health service improvements.

## Introduction

The COVID-19 pandemic continues to pose significant challenges for health systems globally that must respond simultaneously to the needs of people with COVID-19 while maintaining the provision of essential health services.^1^ The continuity of preventive, diagnostic and curative services is critical to reduce avoidable morbidity and mortality.^1^

To respond to the pandemic, health systems reallocated funds, health personnel, infrastructure and equipment from routine services to COVID-19 care and prevention. This prioritisation resulted in a substantial reduction in the capacity to provide essential health services across many countries.^2^ Today, resuming essential health services and managing backlogs of services are critical to improve population’s health. These challenges are complicated by ongoing surges in COVID-19 cases and emerging variants.^1^

Mexico is a middle-income country with a fragmented and underfunded health sector. Since 2013, public health expenditures have declined from 3.1% to 2.8% of the Gross Domestic Product (GDP) as of 2021.^3^

The national response to the COVID-19 pandemic comprised a sample-based sentinel surveillance model with limited COVID-19 testing and containment policies, including the closure of schools and non-essential businesses and recommendations to the public for physical distancing and sanitary measures.^4^ Above all, Mexico prioritised hospital care for severe COVID-19 cases while limiting the provision of essential health services.^4^ As of 28 December 2021, Mexico had one of the world’s lowest COVID-19 testing rates (93 772 tests per million people) and the third-highest case fatality rate (7.6%).^5^ Equally important, Mexico has the highest overall COVID-19 mortality among the Organisation for Economic Co-operation and Development (OECD) member countries with 4456 excess deaths per million people, compared with the OECD average of 1499 per million.^3^ Excess mortality has been higher than registered COVID-19 deaths, indicating underreporting COVID-19 fatalities and an increase in other causes of death.^3^ The high number of adverse outcomes underscored the need to strengthen the COVID-19 response and resume essential health services. To decrease COVID-19 transmission and deaths and contribute to the recovery of essential health services, the Mexican government launched a nationwide COVID-19 vaccination programme in December 2020. By December 2021, 88.0% of Mexican adults had received at least one dose, with 77.8% of adults (over 72 million people) considered fully vaccinated.^6^

The Mexican Institute of Social Security (IMSS) is the largest national public healthcare institution in Mexico that delivers social, economic and health benefits to formal labour market workers and their families through a social insurance model. IMSS covers 68 million people, more than half of Mexico’s population. The institution provides healthcare, including medications, through a nationwide network of primary, secondary and tertiary care medical facilities. Before the COVID-19 pandemic, IMSS was faced with delays in receiving investments for improving hospital infrastructure and for adding new health personnel.^7^ For instance, IMSS has only 2.3 nurses, 1.4 physicians and 0.67 beds per 1000 affiliates. In contrast, OECD member countries report an average of 8.8 nurses, 3.6 physicians and 4.4 hospital beds per 1000 people.^8^

IMSS implemented a COVID-19 strategic response plan congruent with the Ministry of Health recommendations. For instance, IMSS repurposed 23 secondary and tertiary care hospitals to COVID-19 care and 168 hospitals for a combination of COVID-19 care and regular services. In December 2020, 1012 beds had been repurposed. In addition, to face the large wave of COVID-19 cases in Mexico, IMSS hired 34 790 health workers, including medical doctors, nurses, technicians and administrative personnel for COVID-19 care, trained 279 000 health workers on COVID-19 care and created COVID-19 temporary ambulatory care centres.^7^ However, IMSS faced challenges to keep up with essential health services. During the first 9 months of the pandemic, IMSS had significant disruptions in reproductive, maternal, child health and non-communicable disease (NCD) services.^9^ An estimated 8.74 million fewer healthcare visits took place across nine essential health services, such as breast and cervical cancer screening (−79% and −68%), followed by sick child visits (−66%), contraceptive services (−54%), child vaccinations (−36%), diabetes and hypertension care (−32% in both), antenatal care (−27%) and deliveries (−10%).^9^

This commentary describes the National Strategy for Health Services Recovery (NHSR strategy) implemented by IMSS to overcome essential health services disruptions during the second year of the COVID-19 pandemic.

## IMSS National Strategy for Health Services Recovery

In April 2021, IMSS launched the NHSR strategy^10 11^ to resume essential health services delivery. The NHSR strategy was tailored to the context of each state during the pandemic, including the epidemiological situation, the magnitude of disruptions in health services, and the available human resources, and infrastructure.

The NHSR strategy included six components:

1. Reconversion of repurposed COVID-19 hospitals.
  The NHSR strategy included the partial reconversion of repurposed hospitals back to deliver routine essential health services while reserving 30% of hospital capacity for COVID-19 case management.
2. Strengthening COVID-19 prevention measures.
  In keeping with WHO recommendations,^1^ the NHSR strategy strengthened COVID-19 prevention measures. IMSS reinforced passive syndromic surveillance for COVID-19 signs and implemented compulsory testing of symptomatic health workers and those providing COVID-19 care, followed by the quarantine of those who tested positive. However, it did not implement routine COVID-19 testing of health personnel working in facilities that delivered non-COVID care because of the scarcity of financial and human resources and low risk for exposure and transmission.^12^
3. Adjusting governance to prioritise essential health services and optimise service delivery
  The NHSR strategy comprised adjusting governance and coordination mechanisms to prioritise service delivery and facilitate access to essential health services for the affiliates. Family medicine clinics and hospitals working shifts were optimised to return to pre-COVID productivity. Also, IMSS carried out nationwide ‘weekend opening hours’ and monothematic healthcare days such as cataract diagnosis and treatment days to increase access to prioritised services.^11^
4. Implementation of telemedicine services
  IMSS introduced remote digital consultations to enhance access to health services. In June 2021, IMSS started a pilot programme in 18% of its 1500 family medicine clinics. By 1 November 2021, it had provided 154 282 telehealth consultations for COVID-19 patients and those with NCDs.
5. Reinforcement of preventive services and health promotion activities
  IMSS enhanced preventive services and health promotion activities such as national vaccination days, delivery of contraceptives, antenatal care, cervical and breast cancer screening among others. The public was informed continuously about these actions through mass media, phone calls and talks in the waiting rooms, and family medicine consulting rooms.
6. Strengthening monitoring of essential health service provision
  IMSS reinforced the monitoring of essential health service provision in addition to regular monitoring mechanisms already in place to analyse progress in resuming essential health services provision, support catch-up strategies and optimise workforce and resource allocation decisions during the pandemic.

**Figure 1:**
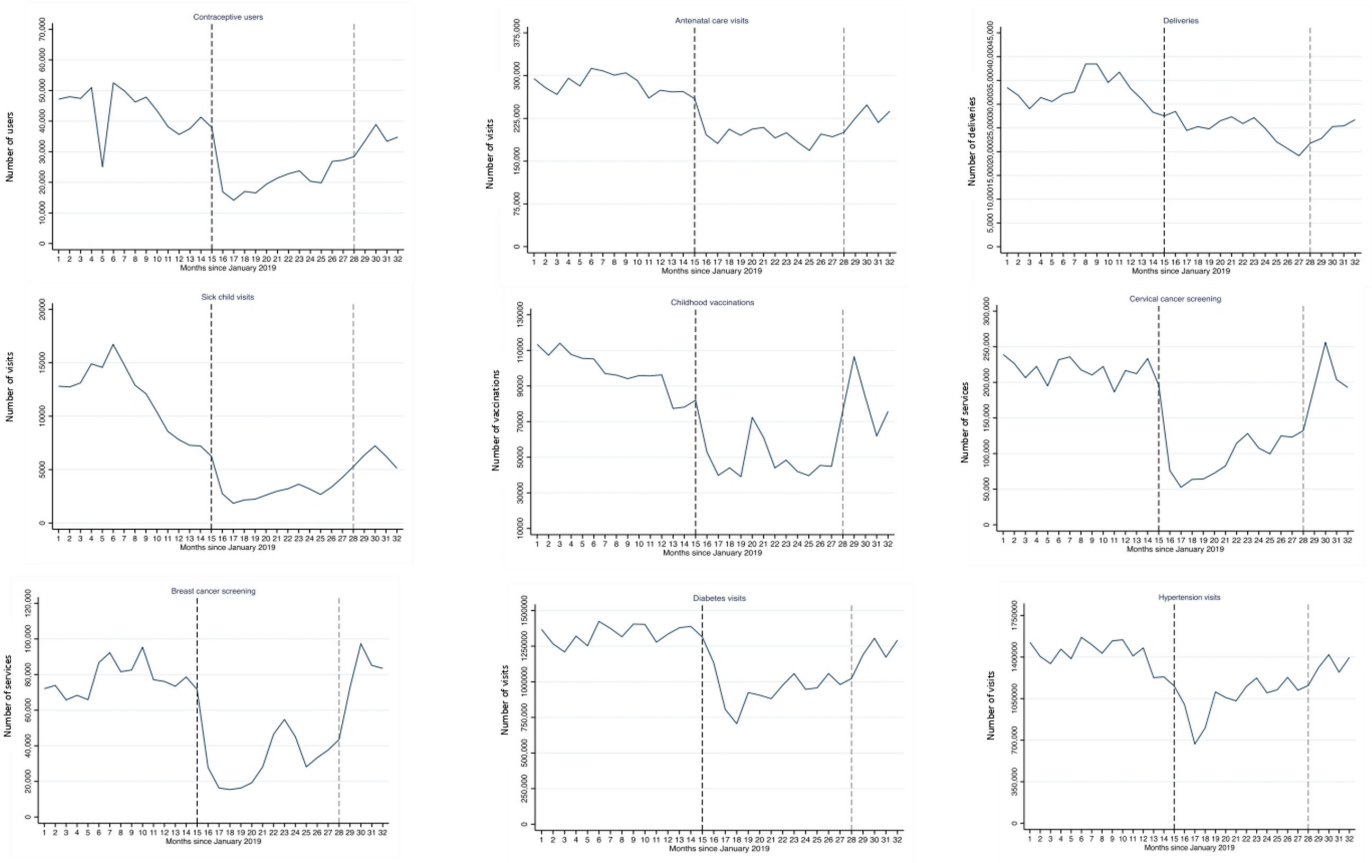
Trends in health service use, Mexican Institute of Social Security, January 2019 to August 2021. Months 1–32 correspond to January 2019–August 2021. The blue lines are the observed service values at the national level. The vertical black dashed line indicates the start of the COVID-19 pandemic in Mexico (1 April 2020). The vertical grey dashed line is the start of the IMSS national strategy for health services recovery (1 April 2021). The definitions of the indicators of services use presented in this figure can be found in the previously published article by Doubova *et al*^9^. For instance, the indicator on children vaccination included information on Bacillus Calmette-Guérin vaccine; the rotavirus vaccine; the pentavalent vaccine against diphtheria, tetanus, pertussis, polio and Haemophilus influenzae type B; the pneumococcal vaccine; and the measles, mumps and rubella vaccine applied in children 0–9 years.

**Figure 2:**
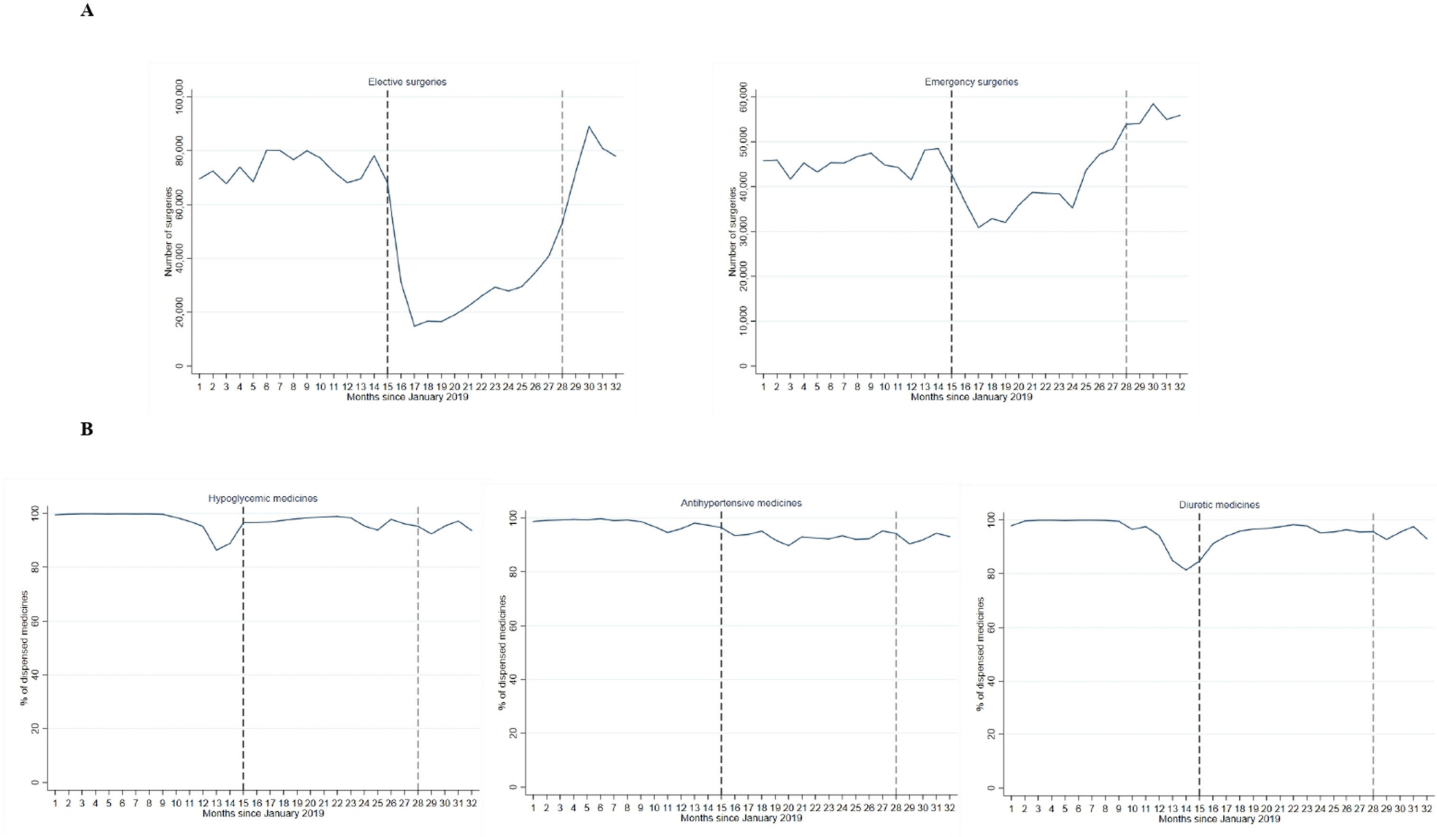
Trends in (A) elective and emergency surgeries and (B) dispensed hypoglycemics, antihypertensives and diuretics, January 2019 to August 2021. Months 1 to 32 correspond to January 2019 to August 2021. the blue lines are the % of dispensed medicines at the national level. The vertical black dashed vertical line indicates the start of the COVID-19 pandemic in Mexico (1 April 2020). The vertical grey dashed vertical line is the start of the IMSS national strategy for health services recovery (1 April 2021). Surgeries are presented as the actual numbers. The proportion of dispensed medications is shown in relation to the total prescribed. I

Figures 1 and 2A show the trends in essential services including selected maternal, child, NCD health services and surgeries from January 2019 to August 2021. The figures illustrate the large declines in most services starting April 2020. By August 2021, most services were still below pre-pandemic levels. Continuous monitoring and further analyses will be needed to identify whether the NHSR strategy was successful in resuming services and tackling the backlog in care for IMSS affiliates.

An adequate supply of medicines is also at the heart of the NHSR strategy. The percentage of medicines dispensed over those prescribed is an important indicator to monitor access to medicines. Chronic patients are sensitive to the disruptions in medicine provision, as they require continuous pharmacological treatment to control their disease and avoid complications. Figure 2B illustrates that during the first year of the pandemic and after the NHSR strategy initiation, there was a continuous provision of hypoglycemics, antihypertensives and diuretics for diabetes and hypertension treatment, since more than 90% of prescribed drugs were dispensed. Yet, there is still room for improvement to achieve the IMSS target of 98%. We chose to present these three groups of treatment, considering that diabetes and hypertension are the leading causes of visits in ambulatory care. However, by presenting information on the dispensing of these three groups of drugs, we cannot assume that the provision of other medicine follows the same pattern.

## Discussion

WHO highlighted the need for health systems to design dynamic and locally relevant strategic responses to effectively address the disruptions in essential health services despite the prolonged course of the COVID-19 pandemic.^1^

Since April 2020, the COVID-19 pandemic put a heavy toll on IMSS, which further unbalanced its already reduced capacity to deliver essential health services. Before the pandemic, between 2012 and 2018, the proportion of IMSS affiliates visiting its health facilities declined from 65.3% to 60.9%, while use of the private sector increased from 30.9% to 33.9%.^13 14^ The COVID-19 pandemic worsened this trend, with only 49% of affiliates using IMSS health facilities in 2020 and nearly the same number, 45% using private sector providers.^15^ Sick child visits, childhood vaccinations and contraceptive users and other health services had a downward trend in 2019, and the COVID-19 pandemic accelerated these negative trends. Other services were also heavily affected by the pandemic such as cervical and breast cancer screening, diabetes and hypertension in-person visits and elective surgeries.

Several factors contributed to declines in essential health services during the COVID-19 pandemic at IMSS. On the supply side, underfunding, shortages in human resources and reallocation of health staff and infrastructure to COVID-19 care reduced the institution’s capacity to deliver essential services.^7 9^ On the demand side, social distancing measures and the population’s fear of contagion contributed to the reduction in users visiting the facilities.^15^ In addition, the continuous surges of the COVID-19 pandemic have been posing a further strain on health services.^7^

The components of the NHSR strategy are congruent with WHO guidelines for maintaining essential health services.^1^ To address the declines in health service provision during COVID-19, IMSS adjusted governance and put in place a series of mechanisms to optimise service delivery. Other strategy components included the reconversion of health facilities, hiring new health personnel, implementing telemedicine services and conducting health promotion activities. IMSS is also monitoring the strategy’s outcomes. An innovative component of the IMSS response included the nationwide ‘weekend opening hours’ strategy. The popularity of this strategy shows that the COVID-19 pandemic can be an important driver for redesigning service delivery.

To strengthen the NHSR strategy, it is advisable to continue reinforcing the supply capacity and implementing communication strategies to inform the public and regain their trust to attend IMSS facilities and overcome the decline in essential health services. The former should address existing health workforce shortages and strengthen the system’s preparedness to manage COVID-19 surges while providing routine health services simultaneously. Additional funding will be required to address gaps in the health workforce and increase the health workforce capacity closer to the OECD average.^7 8^ The temporary staff hired to fight COVID-19 as part of the pandemic’s response is not enough to satisfy the unmet need for health personnel.

Like IMSS, several health systems in different countries started to manage and share their experiences on the strategies to resume essential care. For instance, Nepal, Senegal and Liberia implemented strategies to address disruptions in routine childhood immunisation.^16^ The strategies included infection prevention measures, effective risk communication schemes, mobilisation activities, catch-up campaigns, use of alternative venues for vaccinations, door-to-door vaccinations and actions to identify and reach children with missed doses.^16^ The majority of these strategies were also applied at IMSS, as part of the reinforcement of preventive services and health promotion activities. To overcome the backlogs in children immunisation and avoid vaccine preventable diseases, IMSS should also consider identifying and reaching children with missed doses.

## Conclusion

Before the pandemic, IMSS faced fundamental challenges that resulted in downward trends in essential services. The pandemic accelerated this trend, thus increasing the backlog in services. IMSS is tackling the COVID-19 related disruptions in care through the NHSR strategy. Current service trends signal the need to further strengthen this initiative to resume essential healthcare and overcome the backlog in services. However, utilisation of healthcare and health outcomes at IMSS were not optimal even before the COVID-19 pandemic. Therefore, the NHSR strategy should be the steppingstone for a fundamental change to improve access to and quality of essential services in a large and complex health system.

The lessons learnt from the NHSR strategy could be helpful not only to reinforce the strategy but to restructure the coordination and organisation of services, implement new models of care and accelerate the introduction of digital health technology. However, the Mexican government should support these efforts with adequate funding to enable IMSS to increase the health workforce and invest in infrastructure and medical technology.

Future research should assess whether the NHSR strategy was successful in bringing service levels back to prepandemic trends and overcoming the backlogs in care and provide further evidence for IMSS to strengthen its capacity to deliver health services.

## Data Availability

Data sharing is not applicable.
